# Isolation of Prebiotics from *Artocarpus integer*'s Seed

**DOI:** 10.1155/2021/9940078

**Published:** 2021-07-20

**Authors:** Joel-Ching-Jue Wong, Siew-Ling Hii, Chen-Chung Koh

**Affiliations:** Food Technology Programme, School of Engineering and Technology, University College of Technology Sarawak, 96000 Sibu, Sarawak, Malaysia

## Abstract

There has been a high amount of attention given to prebiotics due to their significant physiological function and health benefits. Prebiotics contain nondigestible compounds that allow specific changes, both in the growth and in the activity of bacteria in the host gastrointestinal tract, that provide benefits upon the host by promoting a healthy digestive system and preventing disease. This study aims at investigating the potential prebiotic activity of bioactive compounds extracted from the seeds of an underutilized indigenous plant *Artocarpus integer* (*A. integer*). The optimum microwave-assisted extraction conditions were a microwave power of 1500 W, extraction time of 180 s, and solvent-to-sample ratio of 1000 : 1. The maximum amount of the total carbohydrate content extracted from *A. integer* was 787 mg/L. The percentage hydrolysis levels of *A. integer* extract in gastric juice at pH 1, 2, 3, and 4 were 6.14%, 7.12%, 8.98%, and 10.23%, respectively. For enzymatic digestion, the percentage of hydrolysis was 0.16% at pH 7. *A. integer* extract was found to support the growth of probiotics such as *L. acidophilus* and *L. casei*. After 72 hours of incubation, *L. acidophilus* achieved 6.96 log_10_ CFU, whereas *L. casei* reached 8.33 log_10_ CFU. The study makes an important contribution to the development of the use of Sarawakian underutilized plants and to the identification of new sources of prebiotic materials to be used in food.

## 1. Introduction

In this globalization era, human societies have become more urban and economic activity is mainly geared towards being manufacturing based rather than being agricultural based. Human lifestyle, diet, and physical activity are greatly affected by this phenomenon. Humans have more leisure time, most of them have the privilege to sample a wider variety of foods, and their lifestyle needs less physical activity. The biodiversity of the human gut microbiota is more likely to be affected by this modern lifestyle, which results in changes of its microbial composition that are associated with increased propensity to a wide range of chronic and noncommunicable diseases (NCD) such as high blood pressure, cardiovascular disease, and type 2 diabetes [1]. The introduction of prebiotics might be a possible way to solve this situation. Many studies on prebiotics have confirmed them to be clinically effective in increasing the number of human gut microbiota to improve human health conditions. According to the International Scientific Association for Probiotics and Prebiotics [2], prebiotics are “substrates that are selectively utilized by host microorganisms conferring health benefits.” The host microorganism is well-known as a probiotic, a selected and viable microorganism that, when introduced in sufficient amounts, beneficially affects the human organisms through their effects in the intestinal tract [3].

The major prebiotics are identified as inulin and oligosaccharides. Prebiotics are often obtained through three different techniques: isolation from natural resources, microbiological synthesis, and enzymatic degradation of polysaccharides [4]. A food ingredient is classified as prebiotic if it fulfils three criteria. First, it must be a nondigestible food which is resistant to gastric acidity, mammalian enzymatic digestion, and intestinal absorption. Second, the food must be susceptible to fermentation by the intestinal microbiota. Third, it must selectively stimulate the growth and/or activity of beneficial microbiota that inhabit the intestine of mammals, including human [5].

As the largest state in Malaysia, Sarawak carries abundant biodiversity and has endemic flora and fauna that are different from the other regions of the country. Plants such as *terung asam*, *dabai*, *cempedak*, and *engkala* have been exploited traditionally by various ethnic and indigenous people living in Sarawak. The crops are highly consumed by Sarawakians and can be considered as a culinary specialty [6]. Nowadays, these plants or crops remain largely underutilized as sustenance for the general population. Underutilized plant species only receive little investment through formal research and development, although they can make a stronger contribution to nutrition, health, income, and sustainability of the environment [7]. However, most of the underutilized plant species are reported as natural sources of bioactive ingredients that possess the potential to be further developed into functional foods. This study aims at investigating the potential of developing underutilized botanical sources into food that helps to maintain the general well-being and prevent diseases. The seed of *chempedak* (*Artocarpus integer*) was selected as an agricultural material for further study.

*A. integer* is the scientific name for the tropical fruit *chempedak*. It is a large and unique composite tropical fruit that belongs to the *Moraceae* family. *A. integer* originates in the forests of equatorial countries in the Indian subcontinent and Southeast Asia countries such as Malaysia, Thailand, Indonesia, Myanmar, and Vietnam, where it grows extensively in the wild [8]. It is locally called *chempedak* and cultivated as a commercial commodity in Malaysia. It offers benefits as a profitable multipurpose crop for producing timber and fruits. In Malaysia, *A. integer* is mainly used for medicinal and culinary purposes. In terms of medicinal use, *A. integer* has been used as a traditional folk medicine in Malaysia for curing diseases such as inflammation, malarial fever and ulcers, abscess, and diarrhoea [9]. In terms of culinary use, *A. integer* is used in its immature and ripe form. Immature fruit is used for making pickles in curry preparation while ripe fruit with golden yellow colour bulbs can be consumed straight. Ripe fruit pulp is eaten fresh or made into different local delicacies, including chutney, jam, jelly, and paste, or preserved as sweets by drying or mixing with sugar, honey, or syrup [10].

The significance of extracting biologically active ingredients of interest from raw materials is generally acknowledged. Extraction represents the primary procedure in the analysis of plants because it is a prerequisite to extract the desired bioactive compounds from the plants. The extracted compounds must undergo further isolation and identification or characterization of its components. The extraction method involved in this study was microwave-assisted extraction (MAE). MAE is one of the innovative techniques of extraction, which has been extensively applied for the isolation, analysis, and quantification of bioactive ingredients. In MAE, microwave energy is used to heat solvents in contact with solid samples or liquid samples (or heat samples, e.g., fresh tissues), promoting the partition of sample-related compounds into the solvent.

## 2. Materials and Methods

### 2.1. Materials

Mature fruits of *A. integer* were obtained from the Central Market, Sibu, Sarawak, Malaysia, and were transported immediately to the laboratory and stored at 4°C until further use. Human salivary *α*-amylase was purchased from Sigma-Aldrich. All chemicals used in this study were of analytical grade unless specified otherwise. *Lactobacillus acidophilus* DSM 20079, *Lactobacillus casei* DSM 20011, and *Escherichia coli* DSM 1103 were purchased from Leibniz-Institut DSMZ GmbH (Braunschweig, Germany). All stock cultures were maintained in sterile glycerol (30% *v*/*v*) at −20°C.

### 2.2. Preparation of *A. intege*r's Fibrous Powder (AIFP)

*A. integer*'s fibrous powder (AIFP) was prepared following Larrauri [11] recommendations for obtaining fibrous powder from the seeds. The fruit was cleaned under running tap water to remove visible dirt. Then, the seeds were obtained by peeling the fruit and removing the pulp. The mass of seeds was determined. The seeds were subjected to a wet-milling process with the ratio of 1 g of seed to 1 mL of water. The juice and pomace from the seeds were separated by a cotton cloth filter. The weight of pomace was recorded. After that, the pomace was cleaned with water for 5 min. The ratio of pomace from seed to water is 1 : 2 (*w*/*v*). After washing, the pomace was filtered by a cotton cloth filter and dried at 50°C for 18 hours. After the drying process, the pomace was ground by blender again to make it into powder form for sieving purpose. The powder was sieved to pass through 250 *μ*m sieve to obtain *A. integer*'s fibrous powder (AIFP). The powder was stored in a polypropylene container at −20°C until further analysis.

### 2.3. Microwave-Assisted Extraction

#### 2.3.1. Experimental Design

In the present study, microwave-assisted extraction method was applied to extract oligosaccharides from *A. integer*'s fibrous powder (AIFP). The two-level full factorial design (FFD) was the first experimental design applied. Three process parameters, i.e., microwave power (*A*, Watt), extraction time (*B*, seconds), and solvent-sample ratio (*C*), were investigated for their effects towards the amount of total carbohydrate (*Y*, mg/L) produced.

Each independent variable was assigned with two levels such as high (+1) and low (−1) levels corresponding to the minimum and maximum values of the factors under evaluation (Table 1).

Table 2 shows a total of twenty-eight (28) experimental runs with eight (8) different combinations of experiments in duplicate and twelve (12) centre points, together with the results of the response of the study. The experimental design was generated and analysed, and the predicted data were calculated using the Design-Expert® Software (version 10, Stat-Ease Inc., Minneapolis, MN, USA).

Following the two-level FFD experimental work, additional experimental runs were performed for the FFD model with significant curvature. This process is known as the augmented central composite design (CCD), which is one of the many designs of the response surface methodology (RSM) technique. The augmented CCD is shown in Table 3. It is a continuation of the two-level FFD by saving time for the experimenter to build another RSM model from scratch.

#### 2.3.2. Extraction and Purification Procedures

Microwave-assisted extraction was performed using a microwave oven with a frequency of 2450 MHz, 34 L capacity, and 1500 W input power. In each of the series of experiments, the *A. integer*'s fibrous powder (AIFP) was mixed with deionised water according to the specified solvent-sample ratio. The extraction was then performed at the respective settings of power and duration (Tables 2 and 3). Following this, the mixture was centrifuged at 4000 rpm, 4°C for 5 min, and the supernatant was mixed with 1 mL of trichloroacetic acid (50% *w*/*v*) to deproteinise the supernatant. After removal of the precipitate, the supernatant was dialysed against deionised water overnight (molecular weight cutoff (MWCO), 500 Da) to remove unwanted low-molecular weight compounds. Nondialysed liquid (extracted sample) was collected and stored at 4°C until further analysis.

### 2.4. *In Vitro* Digestion Studies

#### 2.4.1. Digestion by Gastric Juice

Simulated human gastric juice was prepared by suspending the following chemicals in 1 L of deionised water: 8 g sodium chloride (NaCl), 0.2 g potassium chloride (KCl), 8.25 g disodium phosphate dihydrate (Na_2_HPO_4_.2H_2_O), 14.35 g sodium hydrogen phosphate (NaHPO_4_), 0.1 g calcium chloride dihydrate (CaCl_2_.2H_2_O), and 0.18 g magnesium chloride hexahydrate (MgCl_2_.6H_2_O). The acidity levels of the gastric juice were adjusted to pH 1, 2, 3, and 4 by using 5 M hydrochloric acid (HCl).

One mL of the extracted sample was mixed with 1 mL of simulated human gastric juices of various pH and incubated in a water bath at 37 ± 1°C for 5 hours. After incubation, one mL of the mixture was withdrawn and tested for final reducing sugar content (Section 2.6.2). The 1% *w*/*v* of inulin solution was used as a positive control. The contents of total carbohydrate (Section 2.6.1) and reducing sugar of the extracted sample before the digestion process were also determined.

The percentage of hydrolysis of the extracted sample was calculated by using equation (1) as follows:

(1) $$\begin{array}{c} \%\text{hydrolysis}=\frac{\text{final reducing sugar content}-\text{initial reducing sugar content }}{\text{total carbohydrate content}-\text{initial reducing sugar content}}\times 100\% \end{array}$$

#### 2.4.2. Enzymatic Digestion

*In vitro* enzymatic digestions of extracted samples and inulin (positive control) by using human salivary *α*-amylase were conducted [12]. The enzyme with an activity of 5.2 *μ*mol/mL/min was prepared in 20 mM sodium phosphate buffer (pH 7) to give a final concentration of 0.45 mg/mL.

One mL of enzyme solution was mixed with 1 mL extracted sample. The mixtures were incubated in a water bath at 37 ± 1°C for 5 hours. After incubation, one mL of the mixture was withdrawn and tested for the final reducing sugar content. The 1% *w*/*v* of inulin solution was used as a positive control. The total carbohydrate content and total reducing sugar content of the extracted sample before digestion were also determined. The percentage of hydrolysis of the extracted sample was calculated by using equation (1).

### 2.5. *In Vitro* Fermentation of Extracted Sample

*Lactobacillus acidophilus* DSM 20079, *Lactobacillus casei* DSM 20011, and *Escherichia coli* DSM 1103 were selected to investigate their abilities to ferment the extracted plant materials. The fermentation process was conducted with DeMan, Rogosa, and Sharpe (MRS) broth for the *Lactobacillus* species while tryptic soy (TS) broth was for *E. coli*. Fermentation was carried out in the respective culture broth in which the glucose was replaced by the extracted sample.

After inoculation with the respective bacterial cultures, the media were incubated for up to 72 hours. All incubation, fermentation, and enumeration of *Lactobacillus* species were performed in an anaerobic system.

The viable count of the bacterial cultures was determined by total plate count using MRS agar for the *Lactobacillus* and TS agar for *E. coli.* The result was expressed as log_10_ CFU.

### 2.6. Analytical Procedures

#### 2.6.1. Determination of the Total Carbohydrate Content

The total carbohydrate content was determined by the phenol-sulphuric acid method [13]. One mL of the extracted sample was mixed with 1 mL of 5% phenol solution, and 5 mL concentrated sulphuric acid was added subsequently to the mixture. The mixture was vortexed and incubated in a water bath at 30°C for 30 min before the measurement of absorbance at 490 nm. The amount of total carbohydrate in each sample was obtained using the D-glucose standard curve.

#### 2.6.2. Determination of Reducing Sugar Content

The reducing sugar content was determined by dinitrosalicylic acid (DNSA) assay [14]. One mL of the extracted sample was mixed with 1 mL DNSA reagent. The mixture was heated in a boiling water bath for 15 min. Following this, 0.2 mL of 40% (*w*/*v*) potassium sodium tartrate solution was added to the mixture and 0.8 mL deionised water was added to cool down the mixture. Then, the mixture was vortexed and its absorbance was measured at 575 nm. The D-glucose was used as the standard for determination of the reducing sugar content in the extracted sample.

### 2.7. Statistical Analysis of *In Vitro* Studies

Every experiment was conducted in triplicate. All results were expressed as mean ± standard deviation. Statistical analysis was performed using IBM SPSS version 23 (SPSS Inc., Chicago, IL, USA). Experimental results were subjected to one-way analysis of variance (ANOVA) and the significant difference among the means at 95% confidence limits (*p* < 0.05) was determined by the Tukey test.

## 3. Results and Discussion

### 3.1. Two-Level Full Factorial Design

In the present study, two-level FFD was first employed in the development of three response models, i.e., *A*: microwave power, *B*: extraction time, and *C*: solvent-sample ratio, on the effect of total carbohydrate extracted (*Y*).

In the Design-Expert® Software, the curvature test is placed in front of the ANOVA when centre points are included in the experimental design. The curvature test involves the randomisation of multiple centre points (replicates) throughout the other experimental conditions to get an adequate assessment of whether the actual values measured at this point match what is predicted by the linear model. If the curvature test is significant, a quadratic or higher-order model is needed to model the relationship between the factors and the response.

As shown in Table 4, the curvature test of FFD is significant with a *p* value less than 0.05. Therefore, a response surface model was used as augmentation. A face-centred central composite design (CCD) was chosen as the response surface model, which is comprised of different combinations of six axial points, as shown in Table 3.

### 3.2. Fitting the Central-Composite Design (CCD) Model

Table 5 shows the results of ANOVA for the response surface reduced cubic model conducted at 95% confidence level (*p* = 0.05). The significance of each coefficient of the model was determined using the *F* test and *p* value. The larger the *F* value and the smaller the *p* value, the more significant was the corresponding model term. The *F* value of 112.49 and *p* value that is less than 0.0001 implied the model was very significant. Besides, since the *p* value of “lack of fit” was 0.2170 (*p* > 0.05), “lack of fit” was not significant. A not-significant “lack of fit” indicated that the model fits the actual experimental response data. The ANOVA results confirmed that the model was precise.

The results of regression ANOVA show that the overall mean and standard deviation developed in this model was 234.36 ± 35.91 mg/L (Table 6). The percentage of coefficient of variation (CV %) was 15.32% which represents a minimum variation of data of the model. The coefficient of determination *R*^2^ of 0.9835 indicated an adequately high degree of correlation between experimental and predicted values of the total carbohydrate content. The 98.35% of variation that was observed in the response (total carbohydrate content) can be explained by the model. The difference between adjusted *R*^2^ (0.9603) and predicted *R*^2^ (0.9747) was less than 0.2, which indicated that the adjusted *R*^2^ was in reasonable agreement with the predicted *R*^2^. Based on the results, the model was suggested to be a good simulation of the extraction experiment.

The equation in terms of coded factors (equation (2)) can be used to make predictions about the total carbohydrate content for given levels of each factor. The coded equation is useful for identifying the relative impact of the factors by comparing the factor coefficients. The relative impact of the factors can be observed through the factor coefficients by referring to the coded equation. The factor coefficient of *C* (153.39) is the highest among the three factors. Therefore, factor *C* had the highest impact on the total carbohydrate content. The positive factor coefficient refers to a positive effect and vice versa.

The final equation in terms of coded factors generated by the software was expressed as follows:

(2) $$\begin{array}{c} Y=291.61+86.72A+96.17B+153.39C+90.69AB+81.69AC+76.44BC-75.91A^{2}-89.91B^{2}+86.19ABC. \end{array}$$

The equation in terms of actual factors (equation (3)) can be used to make predictions about the total carbohydrate content for the given levels of each factor. This equation should not be used to determine the relative impact of each factor because the coefficients are scaled to accommodate the units of each factor and the intercept is not the centre of the design space.

The final equation in terms of actual factors generated by the software was expressed as follows:

(3) $$\begin{array}{c} Y=-198.35+0.29A+3.63B+0.21C-2.89x10^{-4}AB-1.28x10^{-4}AC-8.56x10^{-4}BC-1.67x10^{-4}A^{2}-0.016B^{2}+3.78ABC. \end{array}$$

### 3.3. Analysis of Response Surface Plots

**Figure 1:**
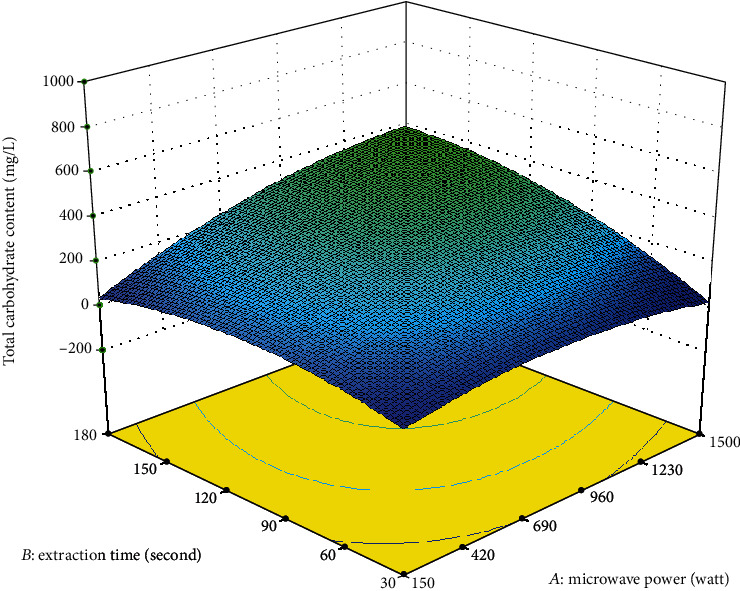
Response surface plot showing the effect of microwave power and extraction time on the total carbohydrate content.

As shown in Figure 1, the amount of total carbohydrate extracted from *A. integer* increased when the microwave power was increased from 150 W to 1230 W, indicating that higher microwave power led to higher extraction of carbohydrate.

Microwave energy acts as a radiation that enhances the lysis of the cell wall. Electromagnetic energy is quickly transferred to the biomolecules, resulting in more power being dissipated inside the solvent, helping to generate molecular movement in the plant materials, and improving the extraction efficiency of polysaccharides at the same time [15]. However, the amount of total carbohydrate extracted from *A. integer* decreased slightly with the further increase in microwave power (above 1230 W). Higher microwave power provides unnecessary energy to the solvent and matrix and drastically disturbs molecular interactions. This disorderly molecular interaction structure as well as higher microwave power may lead to thermal degradation of the polysaccharides, hence the decrease in the amount of total carbohydrate extracted from *A. integer* [16]. Thus, the microwave power should not be too high or else it would be a waste of energy during extraction.

**Figure 2:**
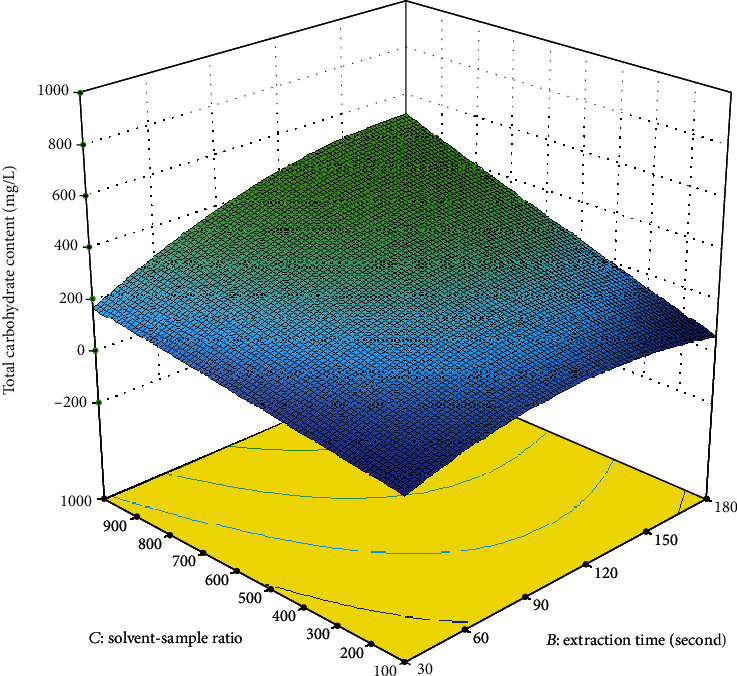
Response surface plot showing the effect of extraction time and solvent-sample ratio on the total carbohydrate content.

The amount of total carbohydrate extracted from *A. integer* increased when the extraction time was increased from 30 s to 150 s, indicating that longer extraction time led to a higher extraction rate of carbohydrate (Figure 2). However, further increase in extraction time decreased the extraction rate. The excessive time exposure in the microwave field may cause polysaccharide degradation [16]. Similar results were observed in the extraction of triterpenoid saponins from *Ganoderma atrum* [17] and extraction of polysaccharides from pumpkin [18].

**Figure 3:**
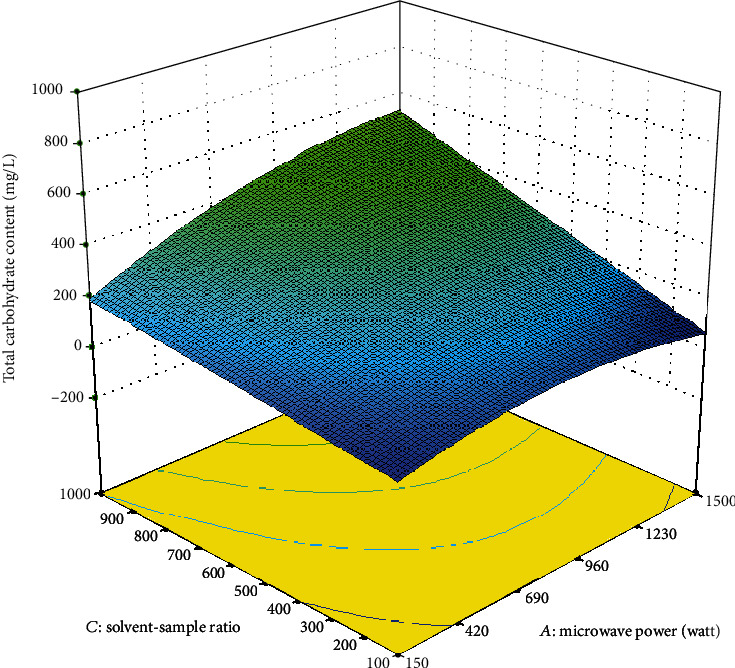
Response surface plot showing the effect of microwave power and solvent-sample ratio on the total carbohydrate content.

In Figure 3, the solvent to sample ratio affects significantly on the amount of total carbohydrate content extracted from *A. integer*. The extraction rate of carbohydrate increased rapidly when the solvent-to-sample ratio was increased. In this study, the volume of the solvent (deionised water) was fixed at 100 mL; thus, the increase in the solvent-sample ratio was also known as the increase in the amount of *A. integer* fibrous powder. Solvent (deionised water) can efficiently absorb microwave energy and enhance the swelling of bioactive compounds, which is favourable to increase the contact surface area between bioactive compounds and the solvent. Thus, the cell walls were ruptured, which resulted in easy release of carbohydrate into the surrounding medium [19].

### 3.4. Verification of the Response Surface Model

To validate the reliability of the response surface model, a verification process was performed based on the desirability value generated by the software. Experiment of verification was conducted using the optimised parameters as follows: 1500 W microwave power, 180 s extraction time, and 1000 : 1 solvent-sample ratio (Table 7). The experiment was conducted in triplicate.

The extracted carbohydrate under predicted optimal conditions is 787.49 mg/L (Table 7) with only 1.2% difference in comparison with the predicted value (797.08 mg/L). The result of the verification test agreed well with the predicted values.

#### 3.4.1. Determination of Degree of Polymerisation

The degree of polymerisation is the number of monosaccharide units in a polysaccharide. The degree of polymerisation was calculated using equation (4).

(4) $$\begin{array}{c} \text{Degree of polymerisation}=\frac{\text{total carbohydrate content}}{\text{reducing sugar content}}. \end{array}$$

In the present study, the oligosaccharides extracted from *A. integer* were having a DP of 16.1. The result indicated that *A. integer* extract has the potential to be considered as a candidate prebiotic since the DP value of the reported prebiotic was within the range of 10 to 200 [20]. Commercial prebiotics such as inulin have a unique range of degrees of polymerisation varying from 2 to 100. The length, composition, and polydispersity of prebiotics depend on the plant species, harvesting time, and extraction and postextraction processes [21].

### 3.5. *In Vitro* Digestion Studies

#### 3.5.1. Digestion by Gastric Juice

Table 8 shows the percentage of hydrolysis of *A. integer* extract and inulin after gastric juice digestion at pH 1 to 4. The percentage levels of hydrolysis of *A. integer* extract after gastric juice digestion at pH 1, 2, 3, and 4 were 6.14%, 7.12%, 8.98%, and 10.23%, respectively, indicating the increase of the reducing sugar content at higher pH conditions. Inulin was used as a positive control in this study, and it was found to have lower gastric juice digestibility.

Food was usually retained in the human stomach where gastric juice (pH 2 to 4) was released within 2 hours. Thus, when *A. integer* extract was consumed, 90% of them were estimated to reach the intestine. Similar results were observed in gastric juice digestion of oligosaccharides from pitaya (dragon fruit) flesh, where 96% of pitaya oligosaccharides were expected to reach the intestine after human consumption [22]. The results of the present study indicated that *A. integer* has the potential to be used as prebiotics due to its ability to resist gastric juice digestion.

#### 3.5.2. Enzymatic Digestion

The enzyme used in the present study was human salivary *α*-amylase. Human salivary *α*-amylase is an enzyme that catalyses the hydrolysis of *α*-glycosidic bonds of polysaccharide starch. When human salivary *α*-amylase reacts with starch, it cuts off the disaccharide maltose. As the reaction progresses, the amount of starch decreases while the amount of maltose increases [23]. The ability of *A. integer* extract and inulin to resist enzymatic digestion was determined based on the percentage of hydrolysis (Table 9). Percentage hydrolysis was calculated using equation (1).

All results are expressed as mean ± standard deviation of three replicates. Results in the same column followed by different superscripts are significantly different (*p* < 0.05).

The percentage levels of hydrolysis of *A. integer* extract and inulin after enzymatic digestion were 0.16% and 0.09%, respectively. Both *A. integer* and inulin showed a very low percentage of hydrolysis after enzymatic digestion, which indicated that they were resistant to enzymatic digestion and could reach the small intestine with less content loss.

According to Johnson and Schmit [24], 30% of the carbohydrates consumed were digested by brush-border enzymes in the small intestine such as isomaltase, glucoamylase, maltase, sucrase, and lactase that hydrolyse *α*-1, 4- and *α*-1, 6-linked glucosaccharides. Therefore, it was estimated that at least 60% of *A. integer* extract consumed would reach the colon since some of them were hydrolysed by gastric juice (10%), by *α*-amylase (0.16%), and by brush-border enzymes in the small intestine (30%). The analysis showed that *A. integer* has the potential to be used as prebiotics due to its ability to resist enzymatic digestion.

In the present study, inulin was found to have similar percentage of hydrolysis with *A. integer* extract. Based on the results, it was estimated at least 69% of inulin would reach the colon after hydrolysis by gastric juice (1.16%), by *α*-amylase (0.09%), and by brush-border enzymes in the small intestine (30%). Similar results were observed by the study conducted by Al-Sheraji, et al. [25], which reported maximum hydrolysis of inulin were 8.02% at pH 7, indicated that more than 60% of inulin reach the colon. According to the ileostomy patient model and intubation model performed by Cummings and Marfarlance [26], 88% of inulin and oligofructose would reach the colon after enzymatic digestion.

### 3.6. Fermentation of *A. integer* Extract by Selected Bacterial Strains

Table 10 shows the growth of *L. acidophilus*, *L. casei*, and *E. coli* in media containing *A. integer* extract and inulin (as positive control). *L. acidophilus* and *L. casei* are well recognised as probiotics that require the carbon source to grow actively. *E. coli* was selected in this study because it is one of the human gut pathogens.

Kolida and Gibson [20] indicated that increments of 0.5 to 1.0 log_10_ in bacterial colonies could be considered as a major shift in the gut microbiota towards a potentially healthier composition of the intestinal microbiota. The presence of the significant growth of *L. acidophilus* and *L. casei* in media containing *A. integer* extract indicated that both *Lactobacillus* strains were able to utilise them as carbon sources and thus conferred potential prebiotic characteristics on them. In addition, the *A. integer* extract had a similar effect to inulin on stimulating the growth of *L. acidophilus* and *L. casei*.

Both *A. integer* extract and inulin were found to support the growth of *E. coli* at the earlier stage of the fermentation process. However, the growth of *E. coli* declined after 48 hours of fermentation, while both *L. acidophilus* and *L. casei* continuously grew up to 72 hours.

Other reported studies have also demonstrated the utilisation of prebiotics by pathogenic bacteria, including the growth of *Clostridia* and *Enterobacteria* in arabinoxylan-substituted media [27], the growth of *Clostridia* in oligosaccharide-substituted media [28], and the growth of *Salmonella* in media containing *Gigantochloa levis* extract [29]. The possibility of these prebiotics utilised by pathogenic bacteria *in vivo* was highly probable based on *in vitro* studies. Furthermore, Shoaf et al. [30] stated that inulin inhibited the adherence of *E. coli* to the epithelial cell surface of tissue culture cells by 30 to 40%, which indicated that inulin does not always inhibit the growth of pathogenic bacteria.

## 4. Conclusion

In summation, *A. integer* is one of the underutilised plant species providing promising potential which is not being capitalised upon. This study served as a starting point for *A. integer* to explore its great potential as a natural resource of prebiotics. *A. integer* extract showed prebiotic properties such as resistant to gastric juice and enzymatic digestion. Approximately 90% of the extract was estimated to reach the intestine after 4 hours of consumption based on the *in vitro* gastric juice digestion study. In addition, the *A. integer* extract was found to be resistant to enzymatic digestion with only 0.16% hydrolysis recorded at pH 7 through the *in vitro* digestion study. The *A. integer* extract was found to support the growth of probiotics such as *L. acidophilus* and *L. casei*. The results of the present study indicated that *A. integer* extract was comparable to the commercial prebiotics inulin. Therefore, *A. integer* extract has the potential to be considered as prebiotic materials and can be used as an ingredient to develop functional foods.

## Figures and Tables

**Table 1:** Independent variables and their levels used in two-level FFD.

Independent variable	Notation	Unit	Low (−1)	High (+1)
Microwave power	*A*	Watt	150	1500
Extraction time	*B*	Seconds	30	180
Solvent-sample ratio	*C*	—	100	1000

**Table 2:** Design matrix of two-level FFD with total carbohydrate (*Y*) as response.

Runs	Experimental factors^a^			Total carbohydrate (mg/L)^b^
	*A*	*B*	*C*	*Y*
1	−1 (150)	−1 (30)	−1(100)	22
2	−1 (80)	+1 (180)	−1 (100)	37
3	−1 (150)	−1 (30)	+1 (1000)	177
4	−1 (150)	+1 (180)	+1 (1000)	167
5	−1 (150)	+1 (180)	+1 (1000)	227
6	0 (825)	0 (105)	0 (550)	415
7	0 (825)	0 (105)	0 (550)	447
8	0 (825)	0 (105)	0 (550)	364
9	+1 (1500)	+1 (180)	+1 (1000)	905
10	+1 (1500)	+1 (180)	+1 (1000)	872
11	+1 (1500)	−1 (30)	−1 (100)	34
12	−1 (150)	−1 (30)	+1 (1000)	219
13	0 (825)	0 (105)	0 (550)	339
14	+1 (1500)	+1 (180)	−1 (100)	94
15	+1 (1500)	−1 (30)	−1 (100)	28
16	−1 (150)	−1 (30)	−1 (100)	36
17	+1 (1500)	+1 (180)	−1 (100)	80
18	+1 (1500)	−1 (30)	+1 (1000)	194
19	−1 (150)	+1 (180)	−1 (100)	97
20	+1 (1500)	−1 (30)	+1 (1000)	170
21	0 (825)	0 (105)	0 (550)	345
22	0 (825)	0 (105)	0 (550)	393

^a^Factors: *A*: microwave power (watts); *B*: extraction time (seconds); *C*: ratio of solvent to sample. Uncoded values are presented in brackets. ^b^Experimental results of the response under investigation.

**Table 3:** Design matrix of augmented CCD.

Runs	Experimental factors^a^			Total carbohydrate (mg/L)^b^
	*A*	*B*	*C*	*Y*
23	−1 (150)	0 (105)	0 (550)	64
24	0 (825)	0 (105)	−1 (100)	41
25	0 (825)	−1 (30)	0 (550)	67
26	+1 (1500)	0 (105)	0 (550)	230
27	0 (825)	+1 (180)	0 (550)	199
28	0 (825)	0 (105)	+1 (1000)	299

^a^Factors: *A*: microwave power (watts); *B*: extraction time (seconds); *C*: solvent-sample ratio. Uncoded units are presented in brackets. ^b^Experimental results of the response under investigation.

**Table 4:** ANOVA of two-level FFD.

Source	Sum of squares^a^ (×10^5^)	*df* ^b^	Mean square^c^ (×10^5^)	*F* value^d^	*p* value, Prob > *F*^e^	Remark
Model	11.24	7	1.605	177.71	<0.0001	Significant
*A*: microwave power	1.216	1	1.216	134.64	<0.0001	
*B*: extraction time	1.598	1	1.598	176.90	<0.0001	
*C*: solvent-sample ratio	3.916	1	3.916	433.47	<0.0001	
*AB*	1.316	1	1.316	145.67	<0.0001	
*AC*	1.068	1	1.068	118.19	<0.0001	
*BC*	0.935	1	0.935	103.49	<0.0001	
*ABC*	1.189	1	1.189	131.57	<0.0001	
Curvature	0.998	1	0.998	110.45	<0.0001	Significant
Pure error	0.090	13	0.090			
Cor total	12.320	21				

^a^Sum of the differences between the squared value of the average value and the overall mean. ^b^Degree of freedom. ^c^Sum of squares divided by *df*. ^d^Ratio of the mean regression sum of squares divided by the mean error sum of squares. ^e^Probability of seeing the observed *F* value if the null hypothesis is true.

**Table 5:** Response surface reduced cubic model for the total carbohydrate content.

Source	Sum of squares (×10^5^)	*df*	Mean square (×10^5^)	*F* value	*p* value, Prob > *F*	Remark
Block	0.543	1	0.543			
Model	13.05	9	1.450	112.49	<0.0001	Significant
*A*: microwave power	1.354	1	1.354	104.99	<0.0001	
*B*: extraction time	1.665	1	1.665	129.10	<0.0001	
*C*: solvent-sample ratio	4.235	1	4.235	328.46	<0.0001	
*AB*	1.316	1	1.316	102.05	<0.0001	
*AC*	1.068	1	1.068	82.80	<0.0001	
*BC*	0.935	1	0.935	72.50	<0.0001	
*A* ^2^	0.190	1	0.190	14.74	0.0013	
*B* ^2^	0.267	1	0.267	20.67	0.0003	
*ABC*	1.189	1	1.189	92.18	<0.0001	
Residual	0.219	17	0.013			
Lack of fit	0.074	4	0.019	1.67	0.2170	Not significant
Pure error	0.149	13	0.011			
Cor total	13.82	27				

**Table 6:** Regression analysis of variance for total carbohydrate content.

Source	Value
SD^a^	35.91
Mean	234.39
CV %^b^	15.32
PRESS^c^	52712.06
*R* ^2^	0.9835
Adjusted *R*^2^	0.9747
Predicted *R*^2^	0.9603
Adequate precision	38.399

^a^Standard deviation. ^b^Percentage of coefficient of variation. ^c^Predicted residual sum of squares.

**Table 7:** Verification of model.

Item	Value
*A*: microwave power	1500 W
*B*: extraction time	180 s
*C*: solvent-sample ratio	1000 : 1
Predicted carbohydrate concentration	797.08 mg/L
Actual carbohydrate concentration	787.49 mg/L
% difference	1.2%

**Table 8:** Percentage hydrolysis by *A. integer* and inulin after gastric juice digestion in various pH.

pH	% hydrolysis	
	*A. integer* extract	Inulin
1	6.14 ± 0.71^a^	0.00 ± 0.00^a^
2	7.12 ± 0.36^ab^	0.72 ± 0.01^b^
3	8.98 ± 0.71^bc^	0.90 ± 0.06^c^
4	10.23 ± 0.36^c^	1.16 ± 0.02^d^

All results are expressed as mean ± standard deviation of three replicates. Results in the same column followed by different superscripts are significantly different (Tukey test, *p* < 0.05).

**Table 9:** Percentage hydrolysis of *A. integer* and inulin after enzymatic digestion and absorption at pH 7.

% hydrolysis	
*A. integer* extract	0.16 ± 0.12^a^
Inulin	0.09 ± 0.04^a^

**Table 10:** Growth of *L. acidophilus*, *L. casei*, and *E. coli* in media containing *A. integer* extract and inulin as the sole carbon source.

Time (hours)	log_10_ CFU/mL					
	*L. acidophilus*		*L. casei*		*E. coli*	
	Extract	Inulin	Extract	Inulin	Extract	Inulin
0	2.46 ± 0.20^a^	2.31 ± 0.25^a^	2.94 ± 0.08^a^	2.98 ± 0.08^a^	2.79 ± 0.02^a^	2.45 ± 0.17^a^
24	6.49 ± 0.25^b^	6.02 ± 0.07^b^	5.36 ± 0.06^b^	5.48 ± 0.20^b^	9.09 ± 0.02^b^	9.11 ± 0.12^bc^
48	6.66 ± 0.07^b^	5.99 ± 0.55^b^	7.45 ± 0.22^c^	7.69 ± 0.17^c^	9.11 ± 0.12^b^	9.28 ± 0.15^b^
72	6.96 ± 0.34^b^	7.05 ± 0.06^c^	8.33 ± 0.17^d^	8.53 ± 0.17^d^	8.90 ± 0.07^c^	8.84 ± 0.22^c^

All results are expressed as mean ± standard deviation of three replicates. Results in the same column followed by different superscripts are significantly different (Tukey test, *p* < 0.05).

## Data Availability

The data used to support the findings of this study are included within the article.
